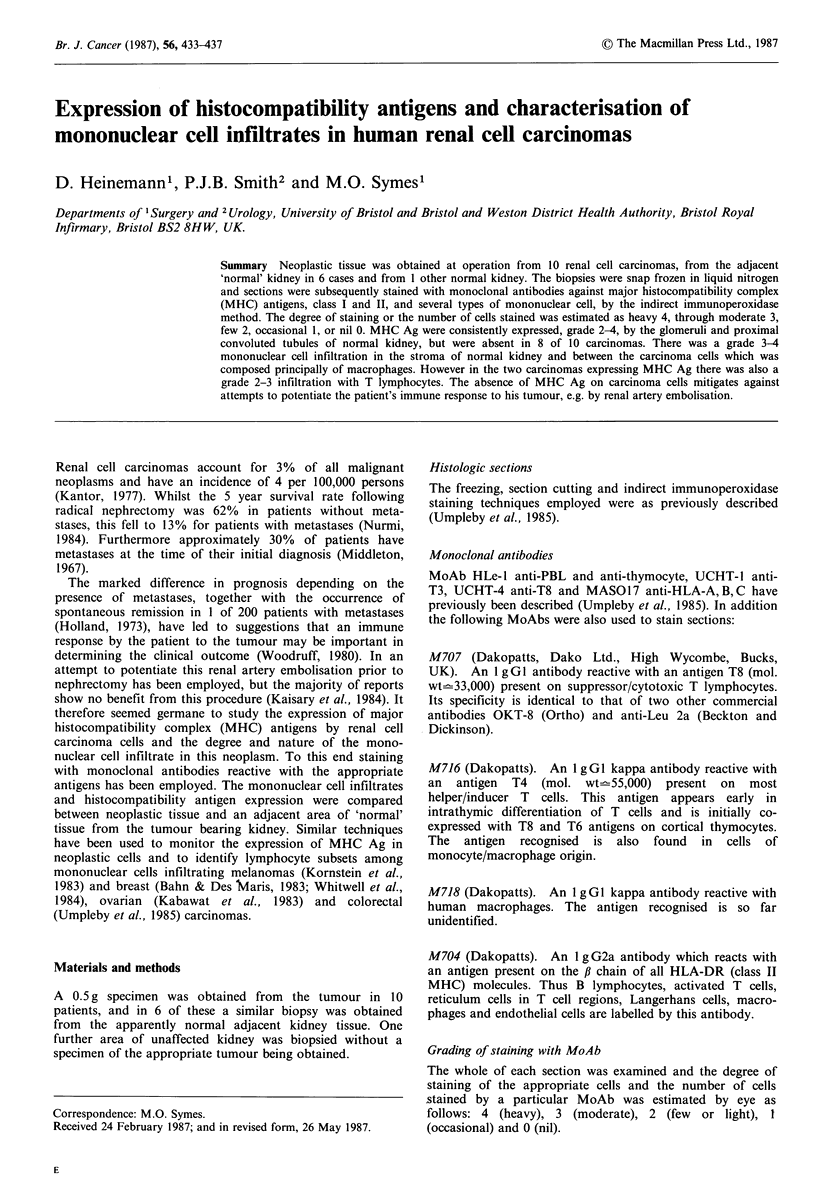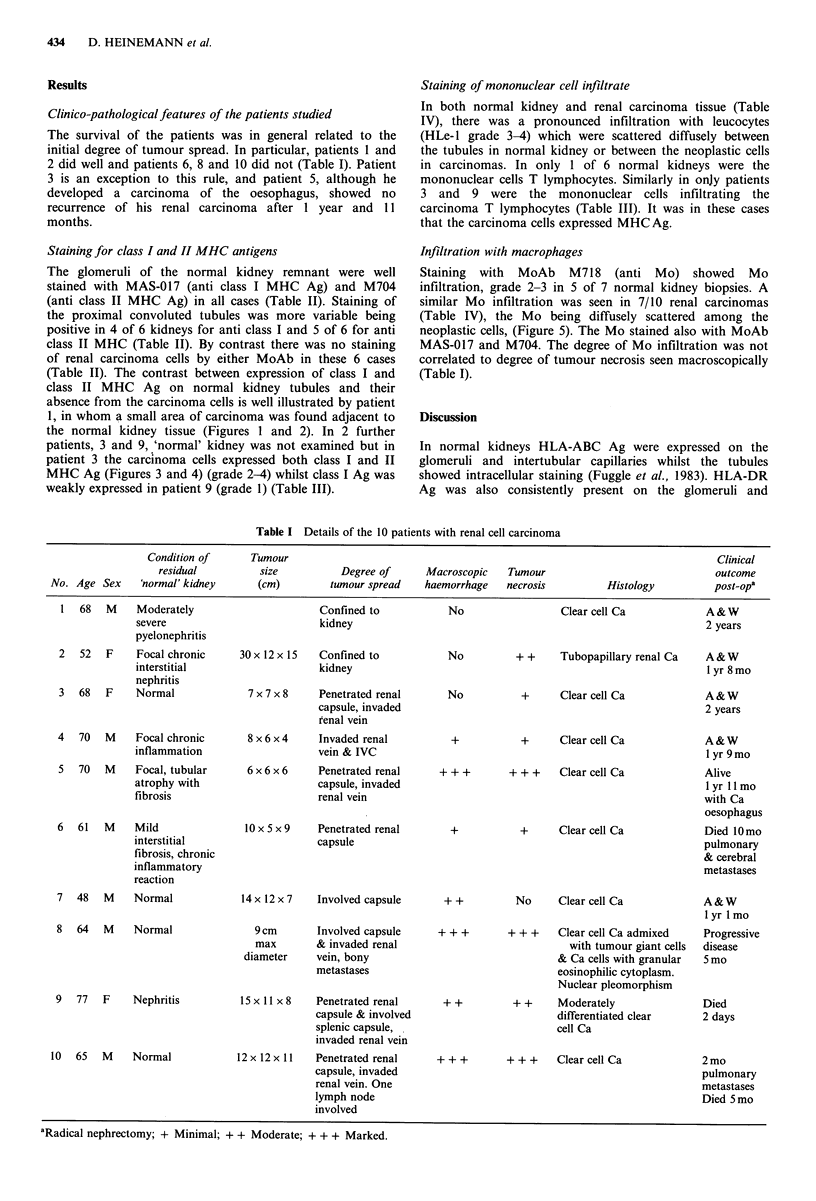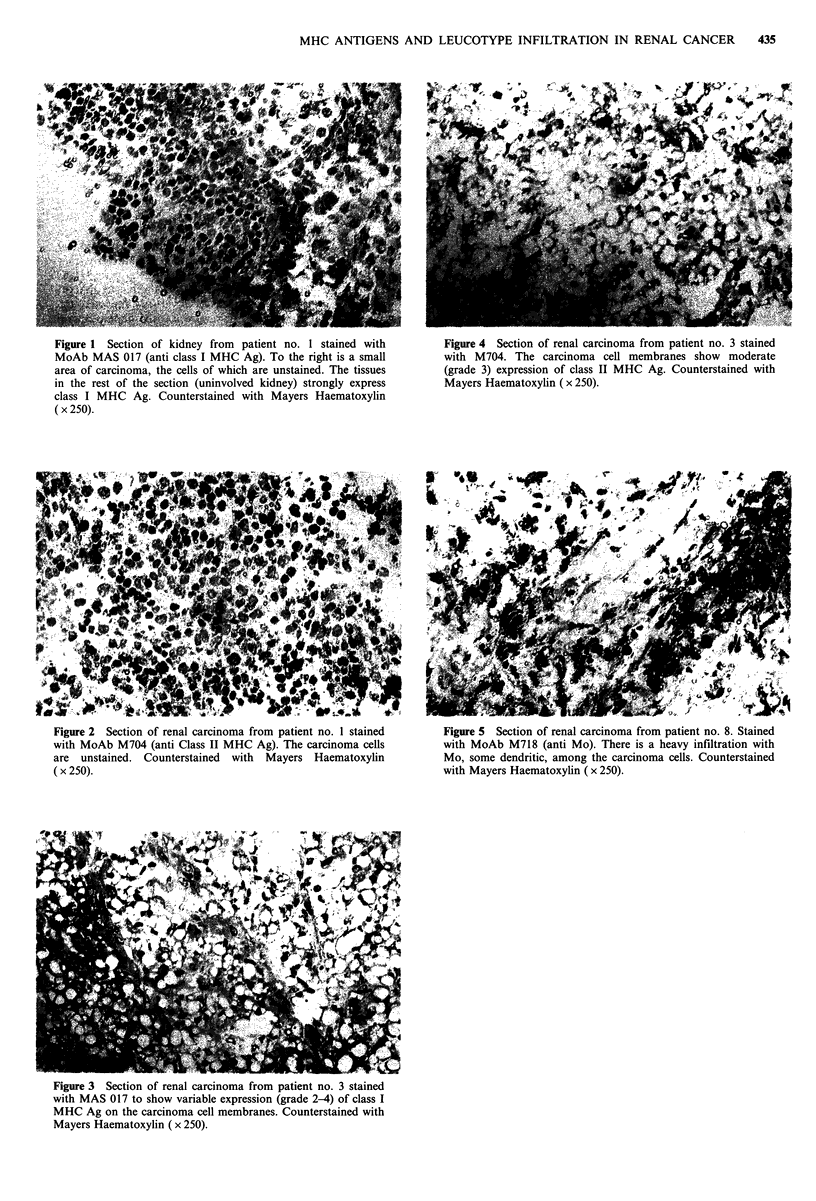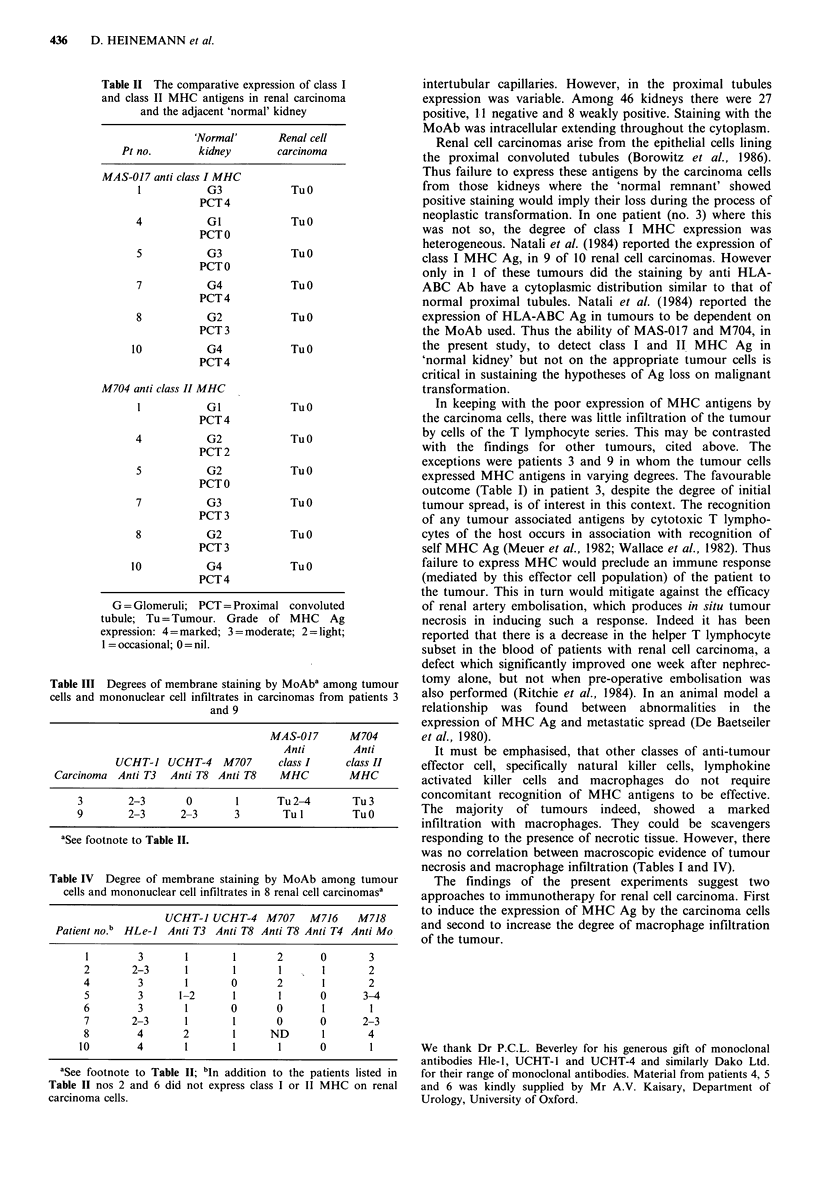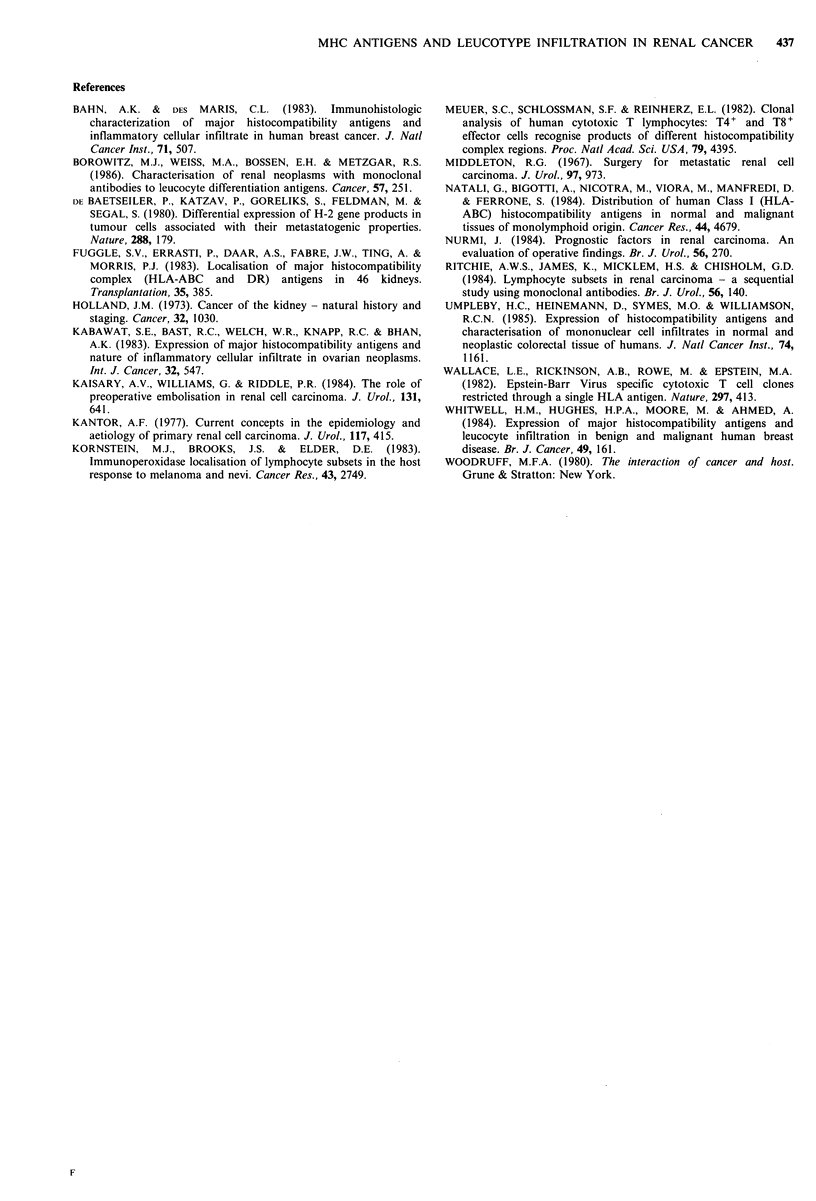# Expression of histocompatibility antigens and characterisation of mononuclear cell infiltrates in human renal cell carcinomas.

**DOI:** 10.1038/bjc.1987.219

**Published:** 1987-10

**Authors:** D. Heinemann, P. J. Smith, M. O. Symes

**Affiliations:** Department of Surgery, University of Bristol, UK.

## Abstract

**Images:**


*Br. J. Cancer* (1987), **56**, 433–437 © The Macmillan Press Ltd., 1987

### Expression of histocompatibility antigens and characterisation of mononuclear cell infiltrates in human renal cell carcinomas

D. Heinemann[1], P.J.B. Smith[2] and M.O. Symes[1]

*Departments of* [1]*Surgery and* [2]*Urology, University of Bristol and Bristol and Weston District Health Authority, Bristol Royal Infirmary, Bristol BS2 8HW, UK.*

**Summary** Neoplastic tissue was obtained at operation from 10 renal cell carcinomas, from the adjacent 'normal' kidney in 6 cases and from 1 other normal kidney. The biopsies were snap frozen in liquid nitrogen and sections were subsequently stained with monoclonal antibodies against major histocompatibility complex (MHC) antigens, class I and II, and several types of mononuclear cell, by the indirect immunoperoxidase method. The degree of staining or the number of cells stained was estimated as heavy 4, through moderate 3, few 2, occasional 1, or nil 0. MHC Ag were consistently expressed, grade 2–4, by the glomeruli and proximal convoluted tubules of normal kidney, but were absent in 8 of 10 carcinomas. There was a grade 3–4 mononuclear cell infiltration in the stroma of normal kidney and between the carcinoma cells which was composed principally of macrophages. However in the two carcinomas expressing MHC Ag there was also a grade 2–3 infiltration with T lymphocytes. The absence of MHC Ag on carcinoma cells mitigates against attempts to potentiate the patient's immune response to his tumour, e.g. by renal artery embolisation.

Renal cell carcinomas account for 3% of all malignant neoplasms and have an incidence of 4 per 100,000 persons (Kantor, 1977). Whilst the 5 year survival rate following radical nephrectomy was 62% in patients without metastases, this fell to 13% for patients with metastases (Nurmi, 1984). Furthermore approximately 30% of patients have metastases at the time of their initial diagnosis (Middleton, 1967).

The marked difference in prognosis depending on the presence of metastases, together with the occurrence of spontaneous remission in 1 of 200 patients with metastases (Holland, 1973), have led to suggestions that an immune response by the patient to the tumour may be important in determining the clinical outcome (Woodruff, 1980). In an attempt to potentiate this renal artery embolisation prior to nephrectomy has been employed, but the majority of reports show no benefit from this procedure (Kaisary *et al.*, 1984). It therefore seemed germane to study the expression of major histocompatibility complex (MHC) antigens by renal cell carcinoma cells and the degree and nature of the mononuclear cell infiltrate in this neoplasm. To this end staining with monoclonal antibodies reactive with the appropriate antigens has been employed. The mononuclear cell infiltrates and histocompatibility antigen expression were compared between neoplastic tissue and an adjacent area of 'normal' tissue from the tumour bearing kidney. Similar techniques have been used to monitor the expression of MHC Ag in neoplastic cells and to identify lymphocyte subsets among mononuclear cells infiltrating melanomas (Kornstein *et al.*, 1983) and breast (Bahn & Des Maris, 1983; Whitwell *et al.*, 1984), ovarian (Kabawat *et al.*, 1983) and colorectal (Umpleby *et al.*, 1985) carcinomas.

### Materials and methods

A 0.5 g specimen was obtained from the tumour in 10 patients, and in 6 of these a similar biopsy was obtained from the apparently normal adjacent kidney tissue. One further area of unaffected kidney was biopsied without a specimen of the appropriate tumour being obtained.

### *Histologic sections*

The freezing, section cutting and indirect immunoperoxidase staining techniques employed were as previously described (Umpleby *et al.*, 1985).

### *Monoclonal antibodies*

MoAb HLe-1 anti-PBL and anti-thymocyte, UCHT-1 anti-T3, UCHT-4 anti-T8 and MASO17 anti-HLA-A, B, C have previously been described (Umpleby *et al.*, 1985). In addition the following MoAbs were also used to stain sections:

*M707* (Dakopatts, Dako Ltd., High Wycombe, Bucks, UK). An 1 g G1 antibody reactive with an antigen T8 (mol. wt≃33,000) present on suppressor/cytotoxic T lymphocytes. Its specificity is identical to that of two other commercial antibodies OKT-8 (Ortho) and anti-Leu 2a (Beckton and Dickinson).

*M716* (Dakopatts). An 1 g G1 kappa antibody reactive with an antigen T4 (mol. wt≃55,000) present on most helper/inducer T cells. This antigen appears early in intrathymic differentiation of T cells and is initially co-expressed with T8 and T6 antigens on cortical thymocytes. The antigen recognised is also found in cells of monocyte/macrophage origin.

*M718* (Dakopatts). An 1 g G1 kappa antibody reactive with human macrophages. The antigen recognised is so far unidentified.

*M704* (Dakopatts). An 1 g G2a antibody which reacts with an antigen present on the $\beta$ chain of all HLA-DR (class II MHC) molecules. Thus B lymphocytes, activated T cells, reticulum cells in T cell regions, Langerhans cells, macrophages and endothelial cells are labelled by this antibody.

### *Grading of staining with MoAb*

The whole of each section was examined and the degree of staining of the appropriate cells and the number of cells stained by a particular MoAb was estimated by eye as follows: 4 (heavy), 3 (moderate), 2 (few or light), 1 (occasional) and 0 (nil).

Correspondence: M.O. Symes.
Received 24 February 1987; and in revised form, 26 May 1987.

### Results

### *Clinico-pathological features of the patients studied*

The survival of the patients was in general related to the initial degree of tumour spread. In particular, patients 1 and 2 did well and patients 6, 8 and 10 did not (Table I). Patient 3 is an exception to this rule, and patient 5, although he developed a carcinoma of the oesophagus, showed no recurrence of his renal carcinoma after 1 year and 11 months.

### *Staining for class I and II MHC antigens*

The glomeruli of the normal kidney remnant were well stained with MAS-017 (anti class I MHC Ag) and M704 (anti class II MHC Ag) in all cases (Table II). Staining of the proximal convoluted tubules was more variable being positive in 4 of 6 kidneys for anti class I and 5 of 6 for anti class II MHC (Table II). By contrast there was no staining of renal carcinoma cells by either MoAb in these 6 cases (Table II). The contrast between expression of class I and class II MHC Ag on normal kidney tubules and their absence from the carcinoma cells is well illustrated by patient 1, in whom a small area of carcinoma was found adjacent to the normal kidney tissue (Figures 1 and 2). In 2 further patients, 3 and 9, 'normal' kidney was not examined but in patient 3 the carcinoma cells expressed both class I and II MHC Ag (Figures 3 and 4) (grade 2–4) whilst class I Ag was weakly expressed in patient 9 (grade 1) (Table III).

### *Staining of mononuclear cell infiltrate*

In both normal kidney and renal carcinoma tissue (Table IV), there was a pronounced infiltration with leucocytes (HLe-1 grade 3–4) which were scattered diffusely between the tubules in normal kidney or between the neoplastic cells in carcinomas. In only 1 of 6 normal kidneys were the mononuclear cells T lymphocytes. Similarly in only patients 3 and 9 were the mononuclear cells infiltrating the carcinoma T lymphocytes (Table III). It was in these cases that the carcinoma cells expressed MHC Ag.

### *Infiltration with macrophages*

Staining with MoAb M718 (anti Mo) showed Mo infiltration, grade 2–3 in 5 of 7 normal kidney biopsies. A similar Mo infiltration was seen in 7/10 renal carcinomas (Table IV), the Mo being diffusely scattered among the neoplastic cells, (Figure 5). The Mo stained also with MoAb MAS-017 and M704. The degree of Mo infiltration was not correlated to degree of tumour necrosis seen macroscopically (Table I).

### Discussion

In normal kidneys HLA-ABC Ag were expressed on the glomeruli and intertubular capillaries whilst the tubules showed intracellular staining (Fuggle *et al.*, 1983). HLA-DR Ag was also consistently present on the glomeruli and

**Table I** Details of the 10 patients with renal cell carcinoma

| *No.* | *Age* | *Sex* | *Condition of residual 'normal' kidney* | *Tumour size (cm)* | *Degree of tumour spread* | *Macroscopic haemorrhage* | *Tumour necrosis* | *Histology* | *Clinical outcome post-op*[a] |
|---|---|---|---|---|---|---|---|---|---|
| 1 | 68 | M | Moderately severe pyelonephritis | | Confined to kidney | No | | Clear cell Ca | A & W 2 years |
| 2 | 52 | F | Focal chronic interstitial nephritis | 30 x 12 x 15 | Confined to kidney | No | + + | Tubopapillary renal Ca | A & W 1 yr 8 mo |
| 3 | 68 | F | Normal | 7 x 7 x 8 | Penetrated renal capsule, invaded renal vein | No | + | Clear cell Ca | A & W 2 years |
| 4 | 70 | M | Focal chronic inflammation | 8 x 6 x 4 | Invaded renal vein & IVC | + | + | Clear cell Ca | A & W 1 yr 9 mo |
| 5 | 70 | M | Focal, tubular atrophy with fibrosis | 6 x 6 x 6 | Penetrated renal capsule, invaded renal vein | + + + | + + + | Clear cell Ca | Alive 1 yr 11 mo with Ca oesophagus |
| 6 | 61 | M | Mild interstitial fibrosis, chronic inflammatory reaction | 10 x 5 x 9 | Penetrated renal capsule | + | + | Clear cell Ca | Died 10 mo pulmonary & cerebral metastases |
| 7 | 48 | M | Normal | 14 x 12 x 7 | Involved capsule | + + | No | Clear cell Ca | A & W 1 yr 1 mo |
| 8 | 64 | M | Normal | 9 cm max diameter | Involved capsule & invaded renal vein, bony metastases | + + + | + + + | Clear cell Ca admixed with tumour giant cells & Ca cells with granular eosinophilic cytoplasm. Nuclear pleomorphism | Progressive disease 5 mo |
| 9 | 77 | F | Nephritis | 15 x 11 x 8 | Penetrated renal capsule & involved splenic capsule, invaded renal vein | + + | + + | Moderately differentiated clear cell Ca | Died 2 days |
| 10 | 65 | M | Normal | 12 x 12 x 11 | Penetrated renal capsule, invaded renal vein. One lymph node involved | + + + | + + + | Clear cell Ca | 2 mo pulmonary metastases Died 5 mo |

[a]Radical nephrectomy; + Minimal; + + Moderate; + + + Marked.

**Figure 1** Section of kidney from patient no. 1 stained with MoAb MAS 017 (anti class I MHC Ag). To the right is a small area of carcinoma, the cells of which are unstained. The tissues in the rest of the section (uninvolved kidney) strongly express class I MHC Ag. Counterstained with Mayers Haematoxylin ( × 250).

**Figure 2** Section of renal carcinoma from patient no. 1 stained with MoAb M704 (anti Class II MHC Ag). The carcinoma cells are unstained. Counterstained with Mayers Haematoxylin ( × 250).

**Figure 3** Section of renal carcinoma from patient no. 3 stained with MAS 017 to show variable expression (grade 2–4) of class I MHC Ag on the carcinoma cell membranes. Counterstained with Mayers Haematoxylin ( × 250).

**Figure 4** Section of renal carcinoma from patient no. 3 stained with M704. The carcinoma cell membranes show moderate (grade 3) expression of class II MHC Ag. Counterstained with Mayers Haematoxylin ( × 250).

**Figure 5** Section of renal carcinoma from patient no. 8. Stained with MoAb M718 (anti Mo). There is a heavy infiltration with Mo, some dendritic, among the carcinoma cells. Counterstained with Mayers Haematoxylin ( × 250).

**Table II** The comparative expression of class I and class II MHC antigens in renal carcinoma and the adjacent 'normal' kidney

| *Pt no.* | *'Normal' kidney* | *Renal cell carcinoma* |
|---|---|---|
| *MAS-017 anti class I MHC* | | |
| 1 | G3<br>PCT 4 | Tu 0 |
| 4 | G1<br>PCT 0 | Tu 0 |
| 5 | G3<br>PCT 0 | Tu 0 |
| 7 | G4<br>PCT 4 | Tu 0 |
| 8 | G2<br>PCT 3 | Tu 0 |
| 10 | G4<br>PCT 4 | Tu 0 |
| *M704 anti class II MHC* | | |
| 1 | G1<br>PCT 4 | Tu 0 |
| 4 | G2<br>PCT 2 | Tu 0 |
| 5 | G2<br>PCT 0 | Tu 0 |
| 7 | G3<br>PCT 3 | Tu 0 |
| 8 | G2<br>PCT 3 | Tu 0 |
| 10 | G4<br>PCT 4 | Tu 0 |

G = Glomeruli; PCT = Proximal convoluted tubule; Tu = Tumour. Grade of MHC Ag expression: 4 = marked; 3 = moderate; 2 = light; 1 = occasional; 0 = nil.

**Table III** Degrees of membrane staining by MoAb[a] among tumour cells and mononuclear cell infiltrates in carcinomas from patients 3 and 9

| *Carcinoma* | *UCHT-1 Anti T3* | *UCHT-4 Anti T8* | *M707 Anti T8* | *MAS-017 Anti class I MHC* | *M704 Anti class II MHC* |
|---|---|---|---|---|---|
| 3 | 2–3 | 0 | 1 | Tu 2–4 | Tu 3 |
| 9 | 2–3 | 2–3 | 3 | Tu 1 | Tu 0 |

[a]See footnote to **Table II.**

**Table IV** Degree of membrane staining by MoAb among tumour cells and mononuclear cell infiltrates in 8 renal cell carcinomas[a]

| *Patient no.*[b] | *HLe-1* | *UCHT-1 Anti T3* | *UCHT-4 Anti T8* | *M707 Anti T8* | *M716 Anti T4* | *M718 Anti Mo* |
|---|---|---|---|---|---|---|
| 1 | 3 | 1 | 1 | 2 | 0 | 3 |
| 2 | 2–3 | 1 | 1 | 1 | 1 | 2 |
| 4 | 3 | 1 | 0 | 2 | 1 | 2 |
| 5 | 3 | 1–2 | 1 | 1 | 0 | 3–4 |
| 6 | 3 | 1 | 0 | 0 | 1 | 1 |
| 7 | 2–3 | 1 | 1 | 0 | 0 | 2–3 |
| 8 | 4 | 2 | 1 | ND | 1 | 4 |
| 10 | 4 | 1 | 1 | 1 | 0 | 1 |

[a]See footnote to **Table II**; [b]In addition to the patients listed in **Table II** nos 2 and 6 did not express class I or II MHC on renal carcinoma cells.

intertubular capillaries. However, in the proximal tubules expression was variable. Among 46 kidneys there were 27 positive, 11 negative and 8 weakly positive. Staining with the MoAb was intracellular extending throughout the cytoplasm.

Renal cell carcinomas arise from the epithelial cells lining the proximal convoluted tubules (Borowitz *et al.*, 1986). Thus failure to express these antigens by the carcinoma cells from those kidneys where the 'normal remnant' showed positive staining would imply their loss during the process of neoplastic transformation. In one patient (no. 3) where this was not so, the degree of class I MHC expression was heterogeneous. Natali *et al.* (1984) reported the expression of class I MHC Ag, in 9 of 10 renal cell carcinomas. However only in 1 of these tumours did the staining by anti HLA-ABC Ab have a cytoplasmic distribution similar to that of normal proximal tubules. Natali *et al.* (1984) reported the expression of HLA-ABC Ag in tumours to be dependent on the MoAb used. Thus the ability of MAS-017 and M704, in the present study, to detect class I and II MHC Ag in 'normal kidney' but not on the appropriate tumour cells is critical in sustaining the hypotheses of Ag loss on malignant transformation.

In keeping with the poor expression of MHC antigens by the carcinoma cells, there was little infiltration of the tumour by cells of the T lymphocyte series. This may be contrasted with the findings for other tumours, cited above. The exceptions were patients 3 and 9 in whom the tumour cells expressed MHC antigens in varying degrees. The favourable outcome (Table I) in patient 3, despite the degree of initial tumour spread, is of interest in this context. The recognition of any tumour associated antigens by cytotoxic T lymphocytes of the host occurs in association with recognition of self MHC Ag (Meuer *et al.*, 1982; Wallace *et al.*, 1982). Thus failure to express MHC would preclude an immune response (mediated by this effector cell population) of the patient to the tumour. This in turn would mitigate against the efficacy of renal artery embolisation, which produces *in situ* tumour necrosis in inducing such a response. Indeed it has been reported that there is a decrease in the helper T lymphocyte subset in the blood of patients with renal cell carcinoma, a defect which significantly improved one week after nephrectomy alone, but not when pre-operative embolisation was also performed (Ritchie *et al.*, 1984). In an animal model a relationship was found between abnormalities in the expression of MHC Ag and metastatic spread (De Baetseiler *et al.*, 1980).

It must be emphasised, that other classes of anti-tumour effector cell, specifically natural killer cells, lymphokine activated killer cells and macrophages do not require concomitant recognition of MHC antigens to be effective. The majority of tumours indeed, showed a marked infiltration with macrophages. They could be scavengers responding to the presence of necrotic tissue. However, there was no correlation between macroscopic evidence of tumour necrosis and macrophage infiltration (Tables I and IV).

The findings of the present experiments suggest two approaches to immunotherapy for renal cell carcinoma. First to induce the expression of MHC Ag by the carcinoma cells and second to increase the degree of macrophage infiltration of the tumour.

We thank Dr P.C.L. Beverley for his generous gift of monoclonal antibodies Hle-1, UCHT-1 and UCHT-4 and similarly Dako Ltd. for their range of monoclonal antibodies. Material from patients 4, 5 and 6 was kindly supplied by Mr A.V. Kaisary, Department of Urology, University of Oxford.

### References

BAHN, A.K. & DES MARIS, C.L. (1983). Immunohistologic characterization of major histocompatibility antigens and inflammatory cellular infiltrate in human breast cancer. *J. Natl Cancer Inst.*, **71**, 507.

BOROWITZ, M.J., WEISS, M.A., BOSSEN, E.H. & METZGAR, R.S. (1986). Characterisation of renal neoplasms with monoclonal antibodies to leucocyte differentiation antigens. *Cancer*, **57**, 251.

DE BAETSEILER, P., KATZAV, P., GORELIKS, S., FELDMAN, M. & SEGAL, S. (1980). Differential expression of H-2 gene products in tumour cells associated with their metastatogenic properties. *Nature*, **288**, 179.

FUGGLE, S.V., ERRASTI, P., DAAR, A.S., FABRE, J.W., TING, A. & MORRIS, P.J. (1983). Localisation of major histocompatibility complex (HLA-ABC and DR) antigens in 46 kidneys. *Transplantation*, **35**, 385.

HOLLAND, J.M. (1973). Cancer of the kidney – natural history and staging. *Cancer*, **32**, 1030.

KABAWAT, S.E., BAST, R.C., WELCH, W.R., KNAPP, R.C. & BHAN, A.K. (1983). Expression of major histocompatibility antigens and nature of inflammatory cellular infiltrate in ovarian neoplasms. *Int. J. Cancer*, **32**, 547.

KAISARY, A.V., WILLIAMS, G. & RIDDLE, P.R. (1984). The role of preoperative embolisation in renal cell carcinoma. *J. Urol.*, **131**, 641.

KANTOR, A.F. (1977). Current concepts in the epidemiology and aetiology of primary renal cell carcinoma. *J. Urol.*, **117**, 415.

KORNSTEIN, M.J., BROOKS, J.S. & ELDER, D.E. (1983). Immunoperoxidase localisation of lymphocyte subsets in the host response to melanoma and nevi. *Cancer Res.*, **43**, 2749.

MEUER, S.C., SCHLOSSMAN, S.F. & REINHERZ, E.L. (1982). Clonal analysis of human cytotoxic T lymphocytes: $T4^+$ and $T8^+$ effector cells recognise products of different histocompatibility complex regions. *Proc. Natl Acad. Sci. USA*, **79**, 4395.

MIDDLETON, R.G. (1967). Surgery for metastatic renal cell carcinoma. *J. Urol.*, **97**, 973.

NATALI, G., BIGOTTI, A., NICOTRA, M., VIORA, M., MANFREDI, D. & FERRONE, S. (1984). Distribution of human Class I (HLA-ABC) histocompatibility antigens in normal and malignant tissues of monolymphoid origin. *Cancer Res.*, **44**, 4679.

NURMI, J. (1984). Prognostic factors in renal carcinoma. An evaluation of operative findings. *Br. J. Urol.*, **56**, 270.

RITCHIE, A.W.S., JAMES, K., MICKLEM, H.S. & CHISHOLM, G.D. (1984). Lymphocyte subsets in renal carcinoma – a sequential study using monoclonal antibodies. *Br. J. Urol.*, **56**, 140.

UMPLEBY, H.C., HEINEMANN, D., SYMES, M.O. & WILLIAMSON, R.C.N. (1985). Expression of histocompatibility antigens and characterisation of mononuclear cell infiltrates in normal and neoplastic colorectal tissue of humans. *J. Natl Cancer Inst.*, **74**, 1161.

WALLACE, L.E., RICKINSON, A.B., ROWE, M. & EPSTEIN, M.A. (1982). Epstein-Barr Virus specific cytotoxic T cell clones restricted through a single HLA antigen. *Nature*, **297**, 413.

WHITWELL, H.M., HUGHES, H.P.A., MOORE, M. & AHMED, A. (1984). Expression of major histocompatibility antigens and leucocyte infiltration in benign and malignant human breast disease. *Br. J. Cancer*, **49**, 161.

WOODRUFF, M.F.A. (1980). *The interaction of cancer and host.* Grune & Stratton: New York.